# Torsades De Pointes Electrical Storm Induced by H1N1 in a Patient with KCNH2 Variant of Unknown Significance

**DOI:** 10.1155/2020/8889769

**Published:** 2020-07-23

**Authors:** Bashar Khiatah, Jonathan Dukes, Christina Desai, Amanda Frugoli

**Affiliations:** ^1^Department of Internal Medicine, Community Memorial Hospital, 147 N Brent St, Ventura, CA 93003, USA; ^2^Electrophysiology, Cardiology Associate Medical Group, USA; ^3^Internal Medicine, Community Memorial Hospital, USA; ^4^GME Department, Community Memorial Hospital, USA

## Abstract

This report describes a case of an electrical storm of Torsades De Pointes in a structurally normal heart, following an H1N1 infection in the presence of a genetic variant of unknown significance. The patient was successfully treated with isoproterenol. This case highlights the dilemma of evaluating novel genetic testing results in a clinical setting.

## 1. Clinical Case

A 60-year-old male with an implantable cardioverter-defibrillator (ICD) presented to the emergency room (ER) for being shocked multiple times. He was hemodynamically stable with a blood pressure of 115/72 and a heart rate of 93. He reported fever, muscle aches, runny nose, and a productive cough with white sputum for the last 2 days. The physical exam was unremarkable except for mild erythema in the oropharynx with no exudate.

His past medical history was significant for recurrent Torsades De Pointes (TdP) in association with aspiration pneumonia in 2014 status post-ICD placement; heart catheterization at that time showed completely normal coronary arteries. He denies any family history of cardiac disease in his parents or grandparents.

Differential diagnoses at this point included emergent interrogation of the ICD device which confirmed the diagnosis of TdP. Thus, the differential was for the eliciting factor which was broadly similar to the data found in Table 1.

**Figure 1:**
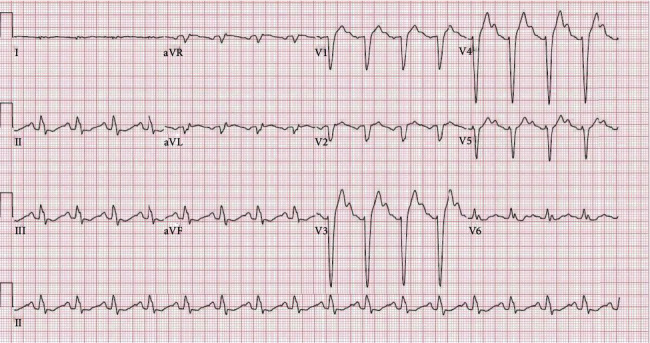
Sinus rhythm with first-degree AV block, left bundle branch block pattern, and delayed QRS transition zone. QTc interval was not prolonged when corrected to the left bundle branch block (LBBB).

Upon further investigations and after interrogating his pacemaker, eleven shocks for ventricular tachycardia (V-Tach) and TdP were confirmed. Electrocardiogram (ECG) showed sinus rhythm (Figure 1), first-degree AV block, left bundle branch block (LBBB) pattern, a wide QRS complex as a result of the left bundle branch block, and a delayed QRS transition zone; QTc interval was not prolonged according to when corrected to the LBBB. (LBBB;QTcH = 458 + 1.75 × (100 − 60) = 528 ms, final QTc = QTcH − 120 × 0.5 = 364 ms, QTc = 468). Mildly elevated troponin is at 0.71 ng/mL with normal electrolyte, complete blood count (CBC), thyroid-stimulating hormone (TSH), arterial blood gas (ABG), and urine drug screen. Influenza screen positive for type A was confirmed later using polymerase chain reaction (PCR) H1N1. Transthoracic echo (TTE), chest X-ray, and myocardial perfusion scan were normal.

**Figure 2:**
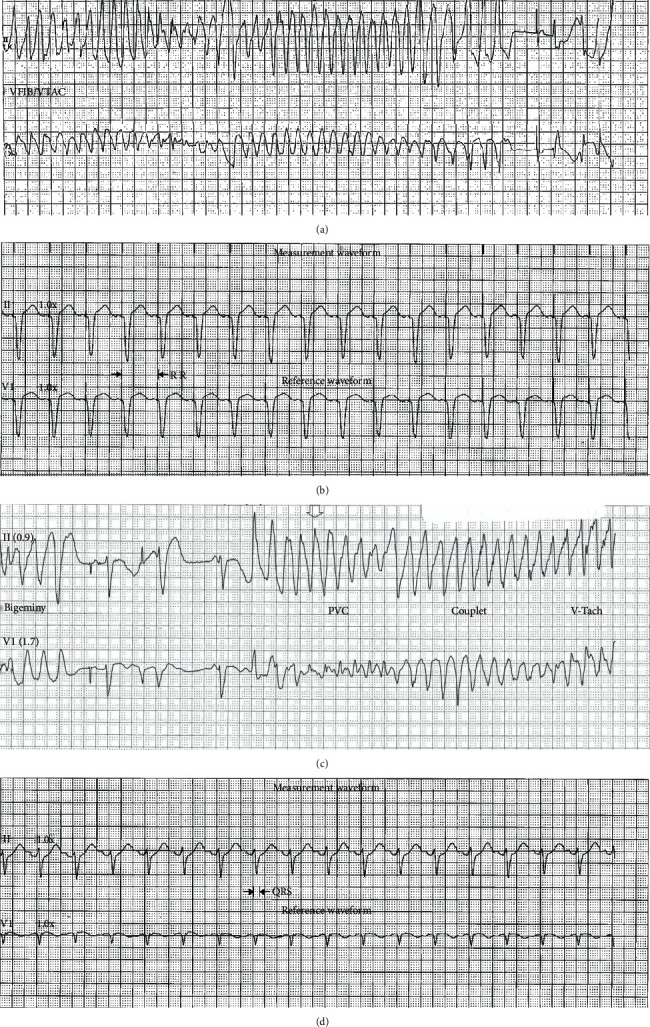
(a) Torsades De Pointes, first episode in the hospital. (b) Setting the ICD at 100 beats per minute. (c) Torsade De Pointes after the replacement of ICD. (d) Dual AV-paced normal ECG by discharge time.

In ER, an amiodarone drip and a bolus were started by the ER physician. Oseltamivir was started, 2 grams of magnesium was given, and he was admitted to the intensive care unit. Overnight, the patient was shocked for multiple runs of TdP and V-Tach as shown in Figure 2(a). Isoproterenol drip was started and titrated to a heart rate of 100-110, which controlled his electrical storm. ICD was set at 100 beats per minute on DDD mode (Figure 2(b)). Over the next 24 hours, he did not have any episodes and was transitioned to quinidine 300 mg every 8 hours. His ICD was at the end of device life, and the patient was scheduled for device replacement. After his device was replaced, he experienced 3 runs of TdP which he was shocked for (Figure 2(c)). Thus, quinidine dose was increased to 648 every 12 hours. After 7 days of initial presentation and 48 hours of episodes free, the patient was discharged home (Figure 2(d)) (QTcH = 420 + 1.75(100 − 60) = 490 ms, final QTc = QTcH − 180 × 0.5 = 400 ms). An outpatient exercise treadmill stress test was scheduled to look for abnormal QT prolongation with exercise and was found negative. Later on (12 weeks after presentation), the genetic cardiac arrhythmia panel revealed; five variants of uncertain significance were detected, one variant of KCNH2 and LDB3 genes, and three of the TTN gene. The patient was called in for a follow-up and switched to nadolol 80 mg qday gradually, since LQTS was a concern despite negative ECG and stress test.

## 2. Discussion

The definition of cardiac electrical storm (ES) is electrical instability of the heart manifesting as recurrent ventricular arrhythmia in a short period of time (>3 in 24 hr). Despite the significant improvement of survival with ICD placement in these patients, ES remains to hold high mortality and morbidity and has a negative impact on long-term outcomes [1]. It is imperative to improve the treatment strategy for ES since the incidence rate of ES is not low (10–28% in patients with an ICD implanted for secondary prevention and 4% for primary prevention) [1]. Given the wide etiologies of Torsades De Pointes (Table 1), it is vital to identify the cause to guide the treatment [1]. Thus, the workup should include ECG, cardiac structural studies with a TTE, myocardial perfusion scan or heart cath, a cardiac enzyme with full electrolyte and basic metabolic panel, thyroid study, blood gas, CBC, blood cultures, chest X-ray, drug screen, home medication review, and finally a cardiac arrhythmia gene panel.

Recognizing the genetic substrate underlying the inherited arrhythmia syndromes has remarkably improved the field of the molecular basis of cardiac electrophysiology, including arrhythmia mechanism and the role of the different ion channels [2, 3]. Genotype-phenotype study relationships, performed mainly for the LQTS, have uncovered the importance of genetic aspects of disease and proved that the patient's management should consider the nature of the gene affected. Any abnormality in ion channel function can result in a disastrous complication that presents as an ECG abnormality and arrhythmias and is usually referred to as cardiac channelopathy. Gene testing for genes coding the cardiac ion channels demonstrated a specific type of inheritable arrhythmogenic disorders that occur in a structurally normal heart and also brought the genetic cardiac channelopathy into focus. These genetic disorders include the LQTS, SQTS, catecholaminergic polymorphic ventricular tachycardia, idiopathic atrial fibrillation, and Brugada syndrome. One of these channels is the potassium channels that are the primary contributors to the repolarization process, which appeared to underlie LQTS in case of dysfunction especially when mutations in genes encoding for pore-forming *α*-subunits (KCNQ1, KCNH2) of these channels are detected [2, 3]. KCNH2 (potassium voltage-gated channel subfamily H member 2), one of many genes, reported having a refractory fever-induced TdP and VF in two related LQT2 patients with the A558P mutation in KCNH2. ECG in these patients showed increased QTc with fever [4]. Currently, more than 1300 KCNH2 variants are recognized in the public genetic archive ClinVar, 5, with almost a third designated as variants of unknown significance [5]. Despite the numerous variants detected for KCNH2, the specific one found in our patient was never reported. Our patient's genetic report showed, a variant of uncertain significant of KCNH2 gene with heterozygous nucleic acid change C.1756 C<G and p.Leu586Val amino acid alteration that has an autosomal dominant inheretance pattern. This have raised many view points; firstly, although the variants in question have not been described previously, a more detailed analysis is warranted as they may be novel disease-causing mutations. Secondly, although the patient has a suspected hereditary LQTS and coding variants in KCNH2, the variants previously have not been described as mutations. Reliably determining whether the variants are causative is therefore not possible. Finally, the patient has a suspected hereditary LQTS consistent with KCNH2 variant, which result in changes to the protein in charge of potassium channel structure; therefore, the variants are likely causative.

Since the ES is an emergency, it is initially managed with the advanced cardiac life support (ACLS) protocol [6], regardless of the etiology of the electrical storm. In advanced settings, the treatment is more etiology specific with pharmacologic choices of amiodarone, quinidine, propranolol, metoprolol, and isoproterenol or interventional therapies with ICD or ablations [7–11]. Neumar et al. compared the combination of IV amiodarone and oral propranolol to the combination of IV amiodarone and oral metoprolol in the management of ES in ICD patients and found amiodarone and propranolol to be superior, safe, and effective in ES management [6]. Isoproterenol showed high efficacy in idiopathic ventricular fibrillation (IVF) patients due to its effectiveness in controlling ventricular fibrillation (VF) and attenuation of the J waves, which showed augmentation prior to the VF onset and was diminished to below the diagnostic level with isoproterenol treatment [9]. In patients with idiopathic VF and Brugada syndrome, the long-term reproducibility of the EP efficacy of quinidine is excellent [10]. In another specific patient population, radiofrequency catheter ablation (RFCA) of refractory V-Tach in patients with myocarditis and RFCA of drug-refractory V-Tach were found to be feasible, safe, and effective [12].

But what if the inducing factor is H1N1 infection without the genetic predisposition or myocarditis? Or the upper respiratory infection with H1N1 in the setting of KCNH2, p.Leu586Val variant is the combination inducing this TdP ES?

In our case, the patient had TdP in 2014 that was believed to be induced by his aspiration pneumonia, and since then, the patient was symptoms free until the recent H1N1 that caused his ES. Oseltamivir was given, which has a safety profile far outweighs the side effects that includes 5-10% nausea, vomiting, and no known arrhythmic side effects that might complicate the patient's situation [13]. While myocarditis has been reported in association with influenza pandemics and interpandemic periods [12], in this case, the lack of different degrees of heart failure (ranging from cardiogenic shock to subtly progressive chronic heart failure), chest pain, bradyarrhythmias, tachyarrhythmias (including sudden cardiac death), negative biochemical markers of myocardial necrosis, normal echocardiographic features, and responding to quinidine is arguing against myocarditis. On the other hand, prodromal symptoms were present and no biopsy was performed.

Also, it has been reported that H1N1 could induce ES in a patient with LQTS which was treated with perfusing magnesium sulfate, increasing the resting pacing rate from 40 to 85 bpm, and increasing propranolol dosage [14]. In contrast, in our patient case with confirmed H1N1 infection in the setting of a negative genetic panel, a successful treatment was achieved with isoproterenol drip and increase pacing rate to 100 bpm with subsequent transition to high dose quinidine. In a follow-up appointment, he was symptom-free for a month with no arrhythmic attacks or pacemaker malfunction. The exercise stress test did not show any abnormal QT prolongation while also acknowledging the sensitivity of this test in unmasking LQTS.

## 3. Conclusion

While more studies are required to understand the effect of H1N1 in patients with genetic variants of uncertain significance, it is worthwhile to consider the potential effect of H1N1 influenza infection in triggering an electrical storm in this population. This may have a great impact as ventricular arrhythmias are a cause of sudden death.

## Figures and Tables

**Table 1:** Causes of polymorphic ventricular arrhythmia [1].

Structural heart diseases	Structurally normal heart	
(i) Ischemic heart diseases (ii) Acute or recent myocardial infarction, prior myocardial infarction (iii) Nonischemic cardiomyopathy (iv) Dilated cardiomyopathy (v) Hypertrophic cardiomyopathy (vi) Arrhythmogenic right ventricular dysplasia/cardiomyopathy (vii) Valvular heart diseases (viii) Corrected congenital heart diseases (ix) Myocarditis (x) Cardiac sarcoidosis (xi) Chagas disease (xii) Metastatic cardiac tumor	Primary causes	Secondary causes
	(i) Idiopathic (ii) Brugada syndrome (iii) Early repolarization syndrome (iv) Long QT syndrome (v) Short QT syndrome (vi) Catecholaminergic polymorphic ventricular tachycardia	(i) Electrolyte abnormalities (ii) Toxic/drug-related (iii) Endocrinologic (iv) Perioperative (v) Iatrogenic (T-wave pacing)
